# Author Correction: Hibernation induces widespread transcriptional remodeling in metabolic tissues of the grizzly bear

**DOI:** 10.1038/s42003-020-0978-1

**Published:** 2020-05-13

**Authors:** Heiko T. Jansen, Shawn Trojahn, Michael W. Saxton, Corey R. Quackenbush, Brandon D. Evans Hutzenbiler, O. Lynne Nelson, Omar E. Cornejo, Charles T. Robbins, Joanna L. Kelley

**Affiliations:** 1 Department of Integrative Physiology and Neuroscience, Washington State University, Pullman, WA 99164 USA; 2 School of Biological Sciences, Washington State University, Pullman, WA 99164 USA; 3 School of the Environment, Washington State University, Pullman, WA 99164 USA; 4 Department of Veterinary Clinical Sciences, College of Veterinary Medicine, Washington State University, Pullman, WA 99164 USA

**Keywords:** Animal physiology, Genetics, Gene expression, Gene regulation, Genomics

Correction to: *Communications Biology* 10.1038/s42003-019-0574-4, published online 13 September 2019.

In the original published version of the article, the Mapping section of the Methods incorrectly read “Trimmed reads were mapped to the brown bear (*Ursus arctos*) reference genome assembly (GCF_000687225.1)^62^ using HISAT2 (version 2.1.0)^63^.” The correct genome assembly reference for *Ursus arctos* is GCF_003584765.1 (reference 25). In addition, the following text was added: “we also mapped the reads to the polar bear (*Ursus maritimus*) reference genome assembly (GCF_000687225.1)^62^ and results were consistent”. The error has been corrected in the HTML and PDF versions of the paper.

