# Supplementary material for: Hibernation induces widespread transcriptional remodeling in metabolic tissues of the grizzly bear
Source: Commun Biol. 2019 Sep 13;2:336. doi: 10.1038/s42003-019-0574-4 (PMC6744400; doi:10.1038/s42003-019-0574-4)
Supplement: Supplementary file 1 — Supplementary Information [file 42003_2019_574_MOESM1_ESM.pdf]

Supplementary Information for

Hibernation induces widespread transcriptional remodeling in metabolic tissues of the grizzly bear

This PDF file includes:

Supplementary Figures 1 to 9
Supplementary Tables 1 to 6

Other supplementary materials for this manuscript include the following:

Supplementary Data 1 to 7 as separate excel files

Supplementary Figures

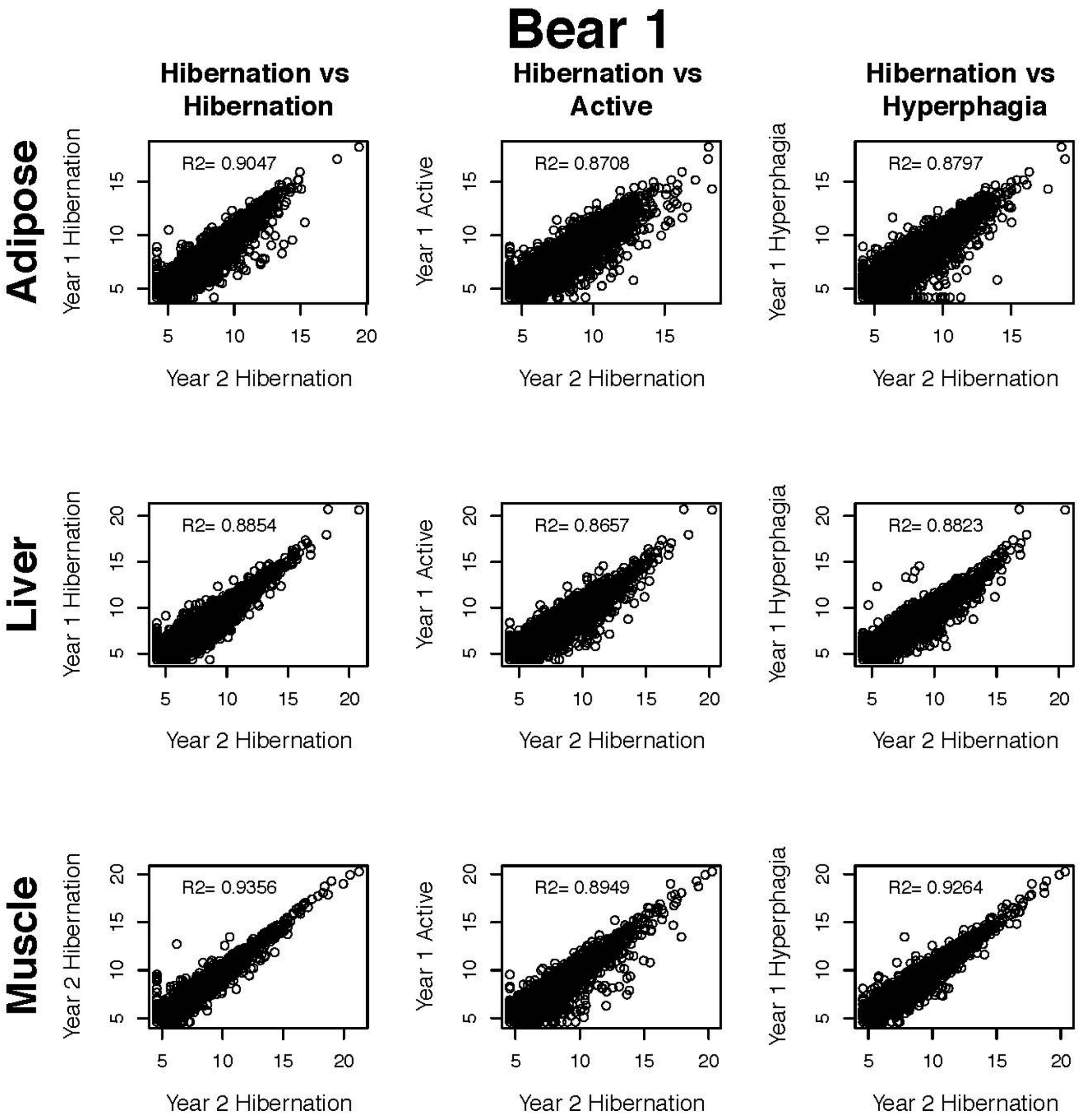

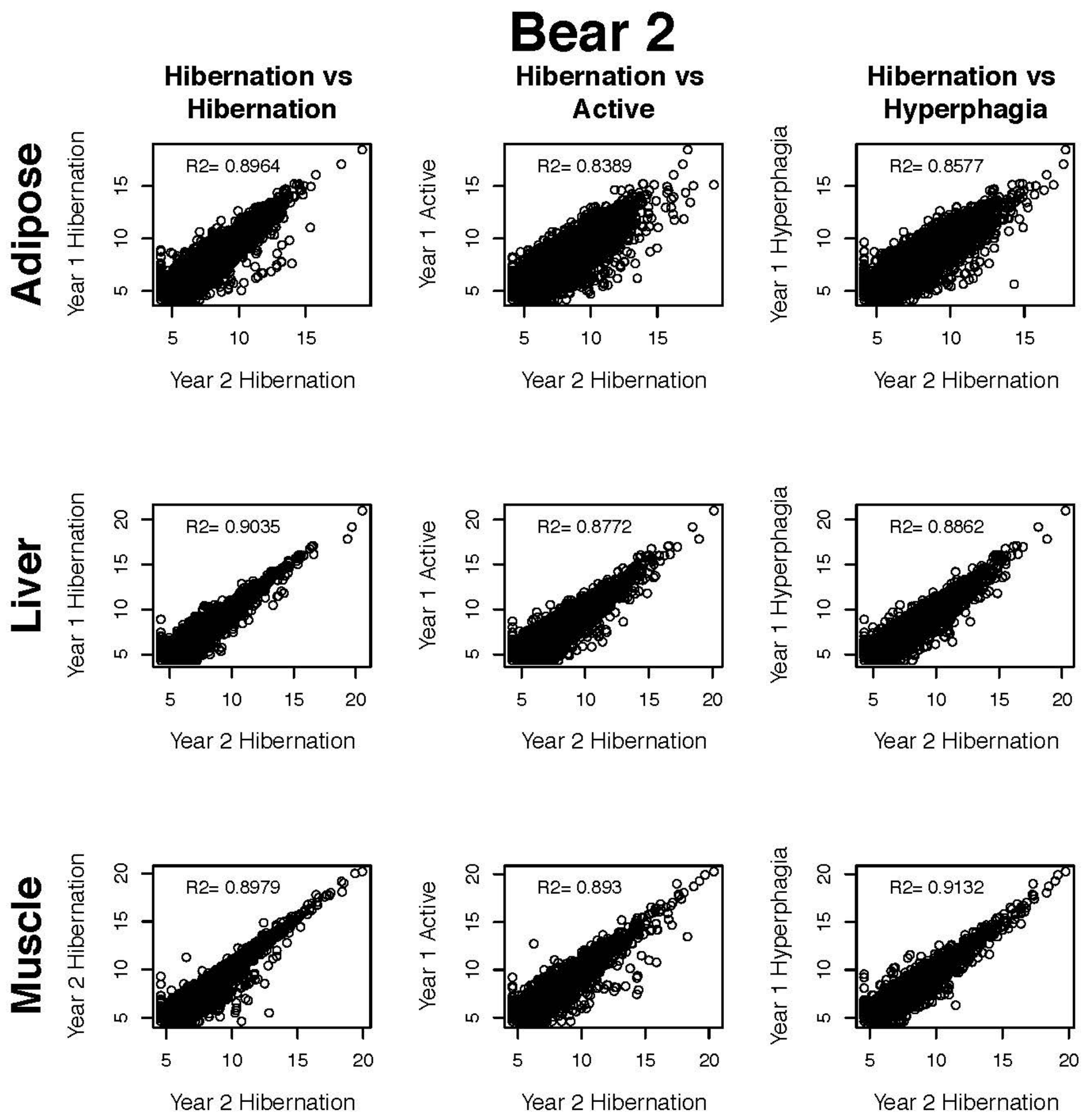

Supplementary Figure 1. Expression of individual genes in metabolic tissues is highly consistent across years.

Normalized counts for all coding genes for two bears sampled in consecutive hibernation seasons. All R^2 values are > 0.85 for all comparisons confirming a high degree of consistency between years.

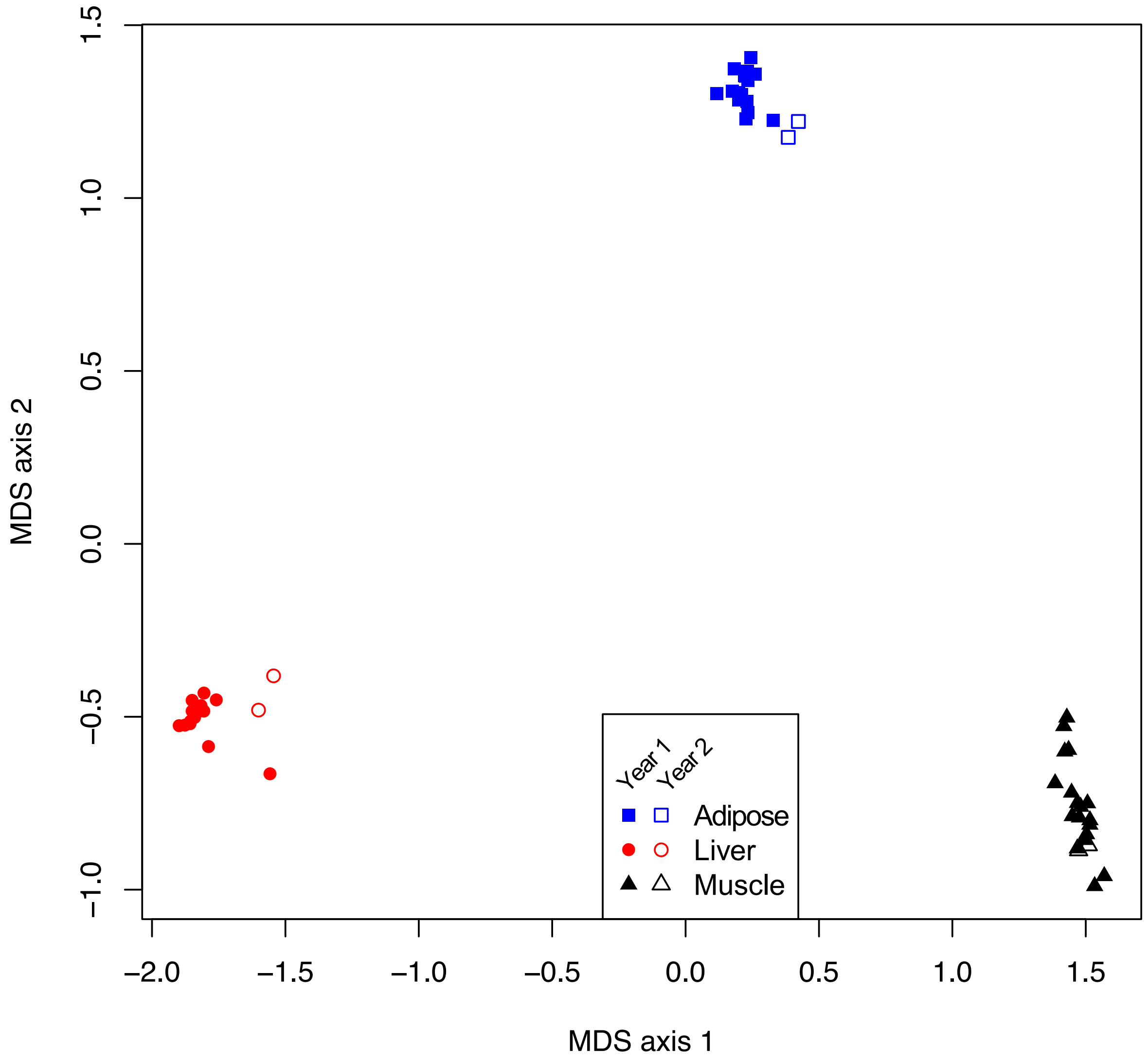

Supplementary Figure 2. Expression differences by tissue

Multidimensional scaling (MDS) plot of all samples shows clear clustering based on tissue type. The MDS analysis included the top 10,000 genes with the largest standard deviations in expression between samples.

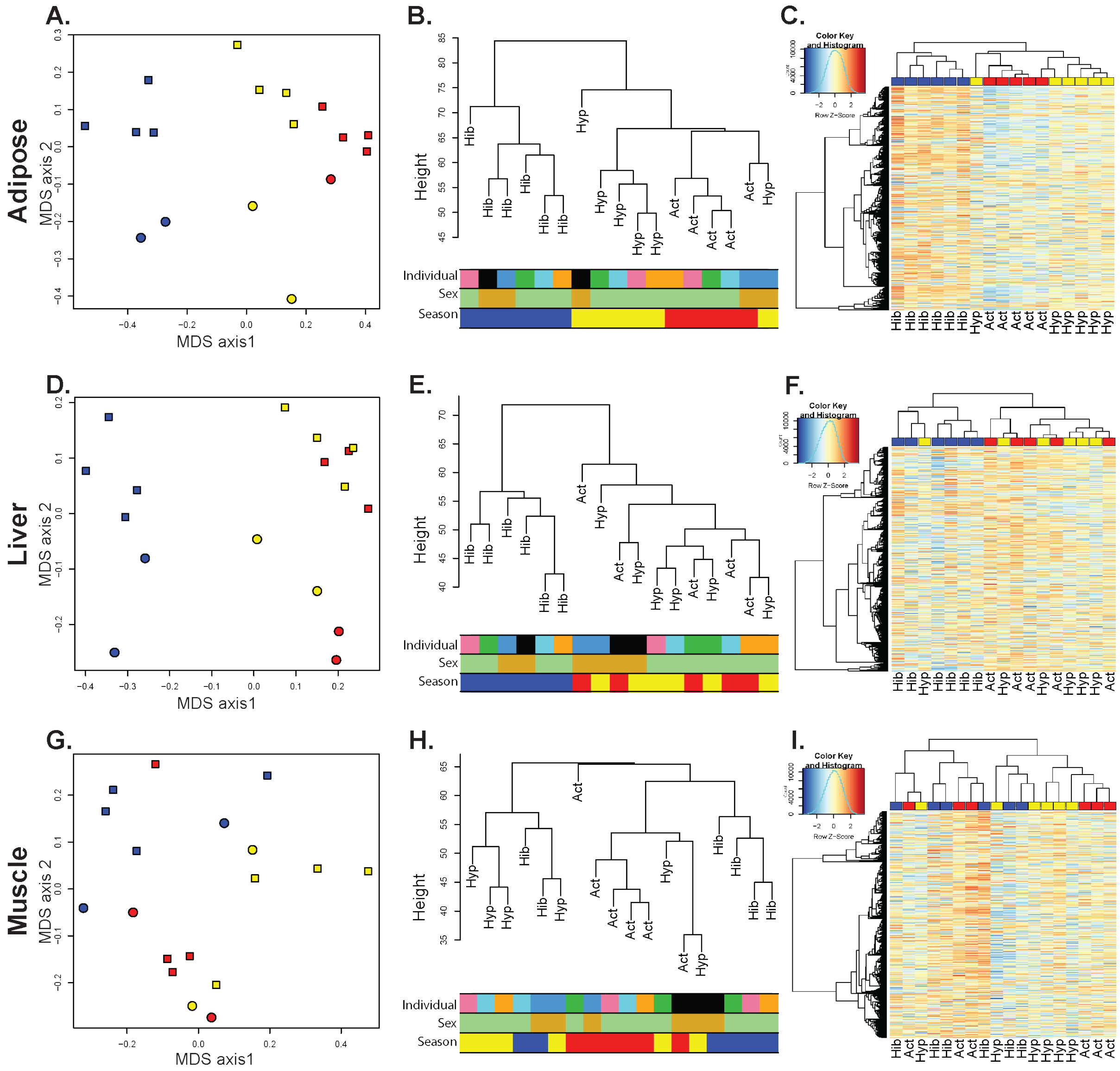

Supplementary Figure 3. Expression differences by tissue and season

Tissue specific multi-dimensional scaling (MDS) plots (**A**, **D**, **G**), dendrograms of samples (**B**, **E**, **H**), and hierarchical clustering of top 10,000 expressed genes (**C**, **F**, **I**). For the MDS plots, the samples are colored by season: active (red), hyperphagia (yellow), hibernation (blue), shapes differentiate females (circle) and male (square). For each dendrogram, three variables were used to visualize clusters (individual, sex, and season); each sample is colored corresponding to the

respective variable (each individual is colored separately, sex: females (orange), males (green), season: active (red), hyperphagia (yellow), hibernation (blue). Dendrograms tips are labeled according to the season sampled. Heatmap of the 10,000 most variable genes inset are Z-scores and distribution of normalized expression levels. There is a clear seasonal separation in adipose and liver, both of which separate hibernation along the first axis of variation (MDS axis 1), moreover, adipose and liver cluster by sex on MDS axis 2 (**A** and **D**). **(G)** Muscle does not display as strong of a seasonal separation along the first axis of variation.

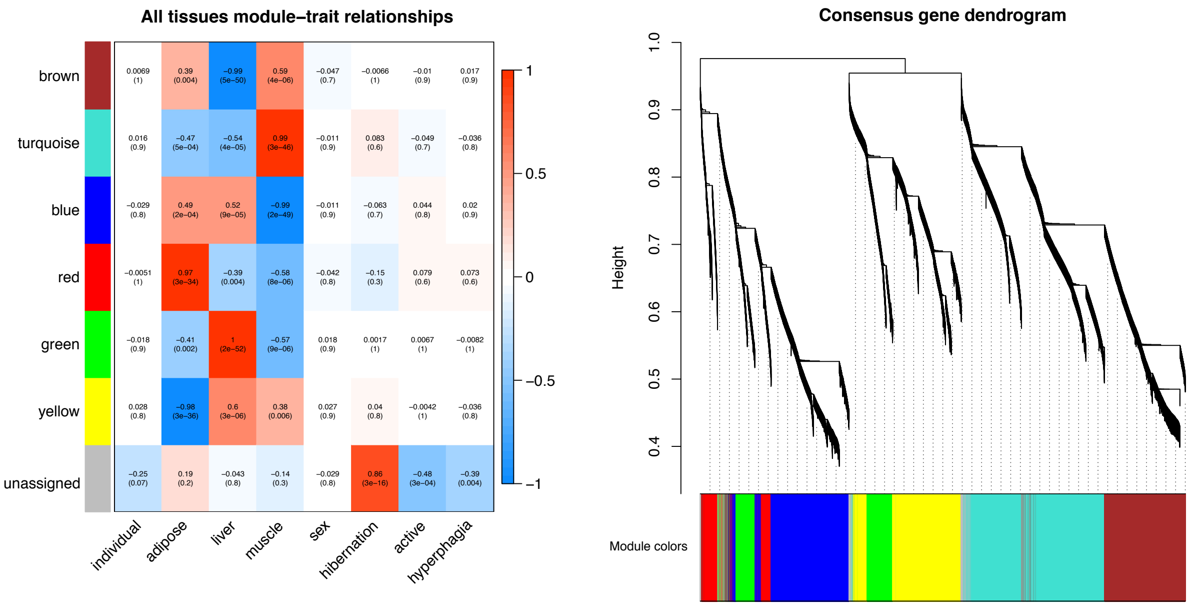

Supplementary Figure 4. Modules from Weighted Gene Co-expression Network Analysis are highly correlated to tissue. **(*left*)** Module-trait relationships. Correlation between module eigenvalues and tissue, sex, and season. Each row represents a module corresponding to a color. Grey represents genes that are not in any module of co-expressed genes (i.e., unassigned). Reported are Pearson correlation coefficients (top of each cell) and *P* values (bottom of each cell, in parentheses). Cell coloration represents the correlation value according to the scale bar on the right. As indicated by the white coloring, there is no significant correlation between sex, individual, or season and module assignment. **(*right*)** Consensus gene dendogram. Average linkage clustering tree based on topological overlap distances in gene expression patterns. Branches of the dendrogram correspond to modules, as shown in the color bar below.

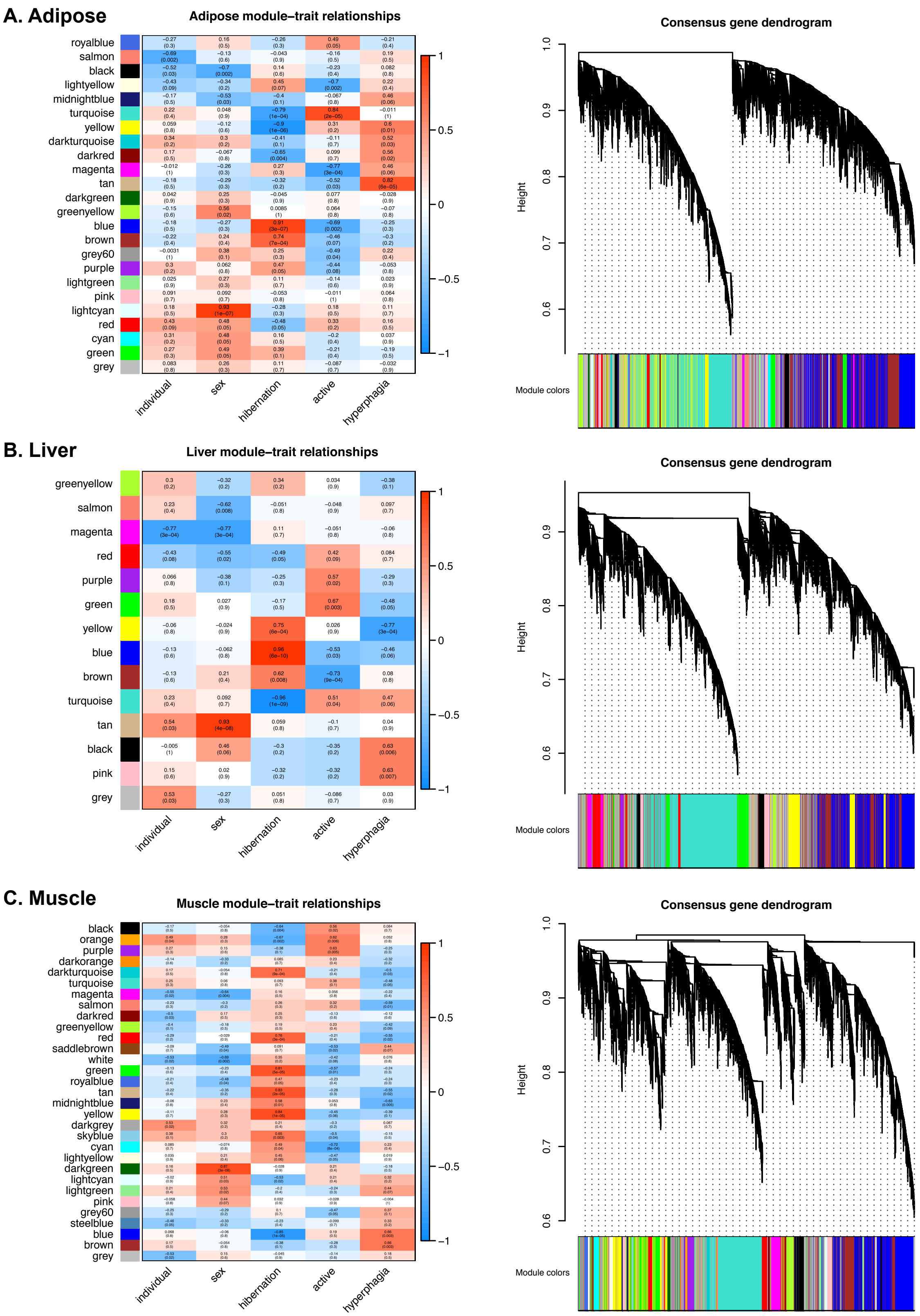
A. Adipose
Adipose module–trait relationships
royalblue
salmon
black
lightyellow
midnightblue
turquoise
yellow
darkturquoise
darkred
magenta
tan
darkgreen
greenyellow
blue
brown
grey60
purple
lightgreen
pink
lightcyan
red
cyan
green
grey
individual
sex
hibernation
active
hyperphagia
Consensus gene dendrogram
Height
Module colors
B. Liver
Liver module–trait relationships
greenyellow
salmon
magenta
red
purple
green
yellow
blue
brown
turquoise
tan
black
pink
grey
individual
sex
hibernation
active
hyperphagia
Consensus gene dendrogram
Height
Module colors
C. Muscle
Muscle module–trait relationships
black
orange
purple
darkorange
darkturquoise
turquoise
magenta
salmon
darkred
greenyellow
red
saddlebrown
white
green
royalblue
tan
midnightblue
yellow
darkgrey
skyblue
cyan
lightyellow
darkgreen
lightcyan
lightgreen
pink
grey60
steelblue
blue
brown
grey
individual
sex
hibernation
active
hyperphagia
Consensus gene dendrogram
Height
Module colors

Supplementary Figure 5. Results of Weighted Gene Co-expression Network Analysis for **(A)** adipose, **(B)** liver, and **(C)** muscle. **(*left*)** Module-trait relationships. Correlation between module eigenvalues and sex, hibernation (versus other two seasons combined), active (versus other two seasons) and hyperphagia (versus other two seasons). Each row represents a module corresponding to a color. Grey module includes genes that are not in any module of co-expressed genes**.** Reported are Pearson correlation coefficients (on the top of each cell) and *P* values (on the bottom in parentheses). Cell coloration represents the correlation value according to the scale bar on the right. **(*right*)** Consensus gene dendogram. Average linkage clustering tree based on topological overlap distances in gene expression patterns. Branches of the dendrogram correspond to modules, as shown in the color bar below. GO terms associated with each colored module can be found in Supplementary Tables S2 (adipose), S3 (liver) and S4 (muscle).

A. Adipose

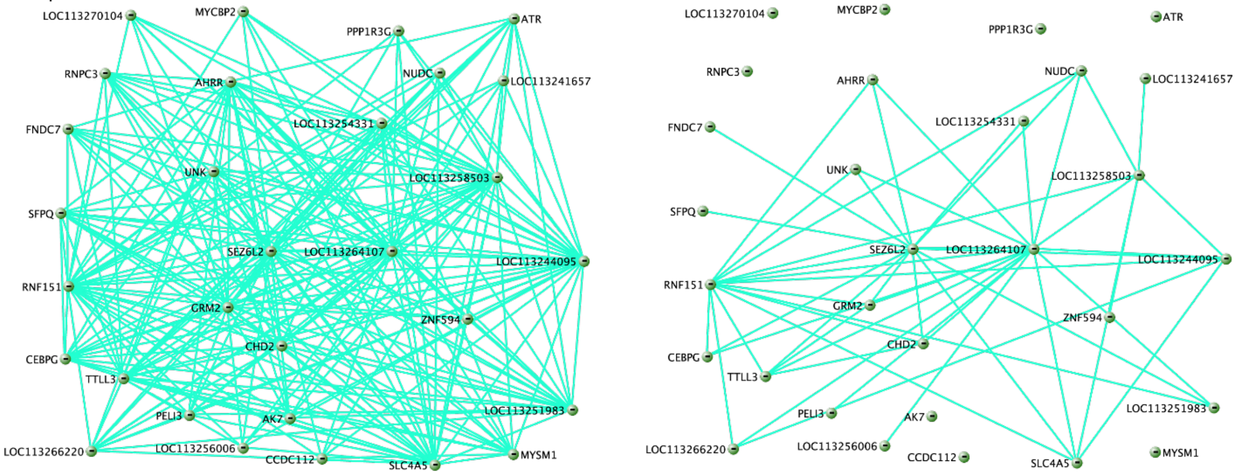

B. Liver

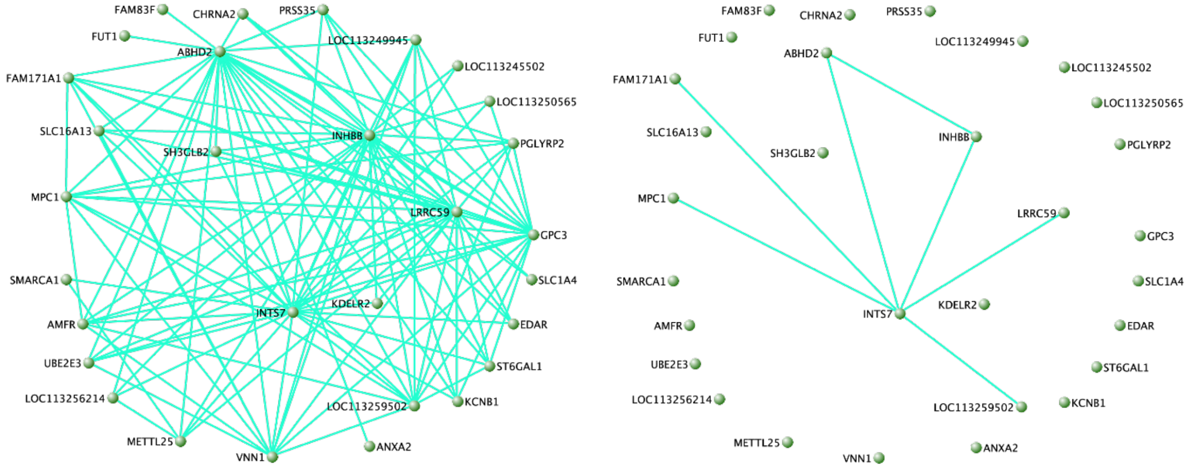

C. Muscle

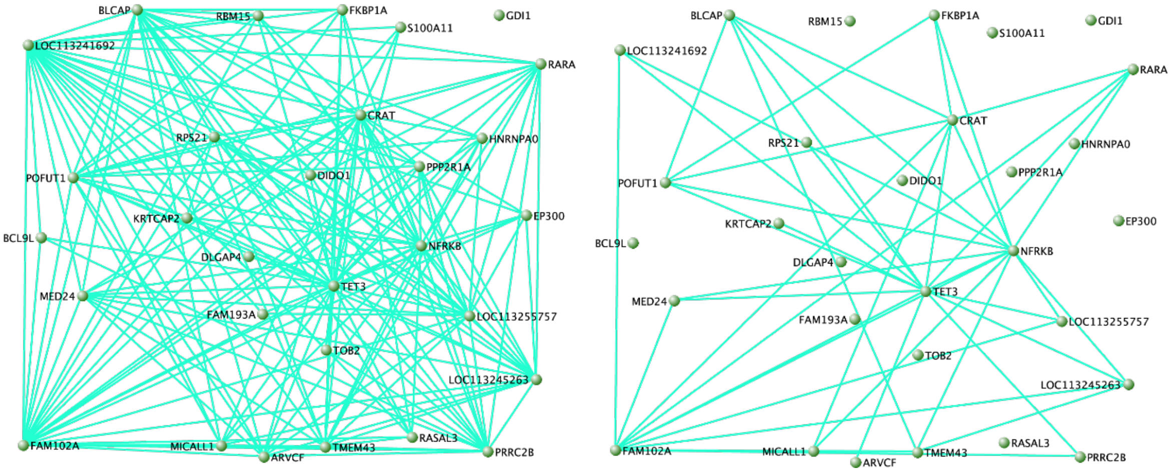

Supplementary Figure 6. Connectivity of top 30 hub genes for modules most significantly positively correlated with hibernation in each tissue. Interaction of gene co-expression patterns by VisANT (version 5.51)[1] in **(A)** Adipose, blue module with connectivity *(left)* 0.28 and *(right)* 0.30. **(B)** Liver, blue module with connectivity *(left)* 0.375 and *(right)* 0.38. **(C)** Muscle, yellow module with connectivity *(left)* 0.2 and *(right)* 0.18. Nodes are labeled with gene symbols from the brown bear annotation.

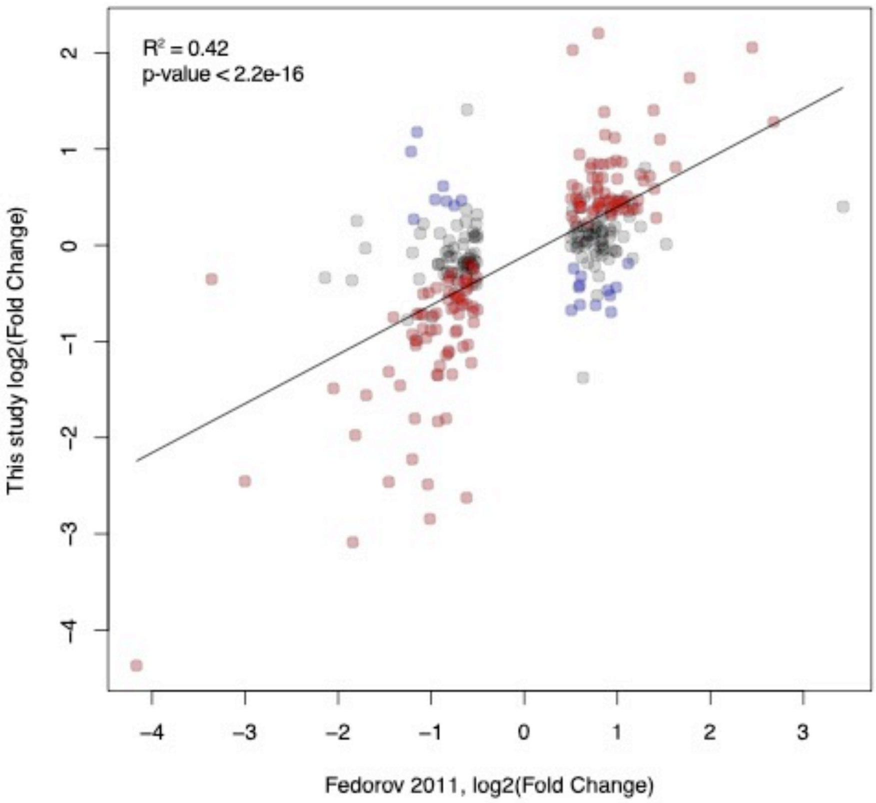

Supplementary Figure 7. Genes in liver that are shared between the current study and Fedorov et al. (2011)[2]. Log_2-fold change was significantly correlated between the two studies (R^2=0.42, p < 2.2e-16). Genes with significant differential expression (FDR<0.05) in both studies that have a log_2-fold change in the same direction (red) or opposite direction (blue) are indicated. Genes that were significantly differentially expressed in Fedorov et al. (2011)[2] but not in our study are in black.

A. Adipose

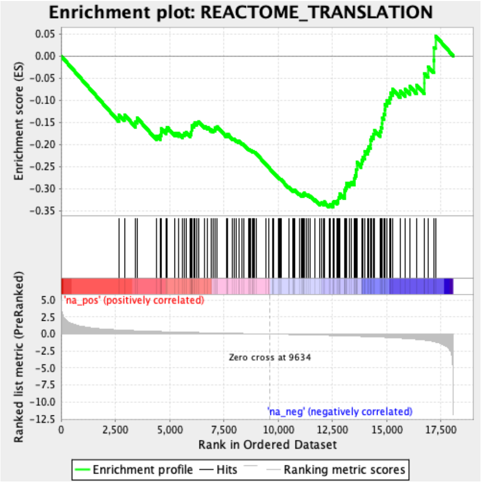

B. Liver

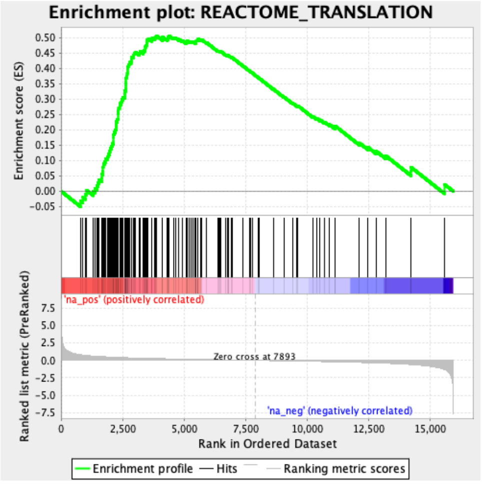

C. Muscle

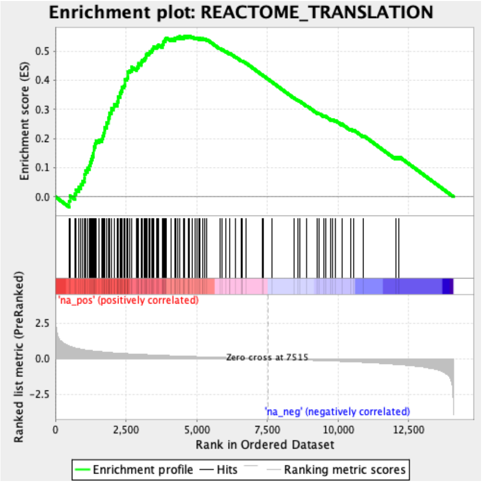

Supplementary Figure 8. Translation gene set enrichment. Enrichment of Reactome Translation Gene Set using Gene Set Enrichment Analysis for **(A)** adipose, **(B)** liver, and **(C)** muscle.

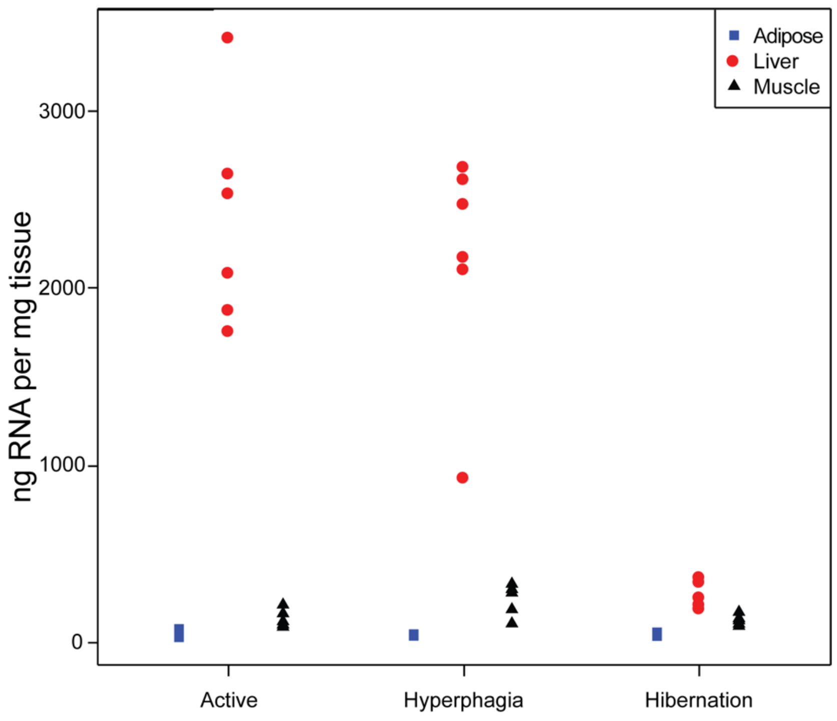

Supplementary Figure 9. Amount of total RNA per season per tissue. Significance measured with Kruskal-Wallis: Adipose p=0.017, Liver p=0.003, Muscle p=0.166. Note the lower amount of total RNA in liver and adipose during hibernation.

Supplementary Tables

Supplementary Table 1. Adipose modules of co-expressed genes, including module color, number of genes in each module, and the number of genes annotated with a BLAST hit in each module. Gene sets were analyzed in Blast2GO to determine whether there were enriched ontologies. The top most significant biological process GO term (selected by lowest FDR) and the largest GO term (selected by the number of genes in the test set) are reported along with number of genes in the test set (Nr Test) and false discovery rate (FDR). Modules with no significant GO enrichment are indicated by -. Modules significantly associated with hibernation are indicated by *.

Adipose Module	# of genes	# of annotated genes	Most Significant GO Term			Largest GO Term		
			GO term	Nr Test	FDR	GO term	Nr Test	FDR
Black	407	339	regulation of multicellular organismal process	181	1.61E-07	biological regulation	291	2.56E-03
Blue*	3575	2706	RNA processing	284	3.57E-11	metabolic process	1964	4.03E-02
Brown*	2553	2242	nucleic acid metabolic process	1138	4.74E-47	cellular process	2029	4.23E-04
Cyan	172	149	system development	102	1.14E-05	developmental process	110	7.89E-05
Dark Green	84	74	leukocyte mediated immunity	37	2.09E-16	response to stimulus	66	1.23E-03
Dark Red*	95	-	-	-	-	-	-	-
Dark Turquoise	78	75	keratinization	31	5.10E-37	multicellular organismal process	62	4.47E-03
Green	466	431	anatomical structure development	294	5.20E-11	metabolic process	342	7.20E-04
Green Yellow	230	183	nucleic acid metabolic process	111	1.52E-07	metabolic process	149	4.67E-02
Grey (unassigned)	2389	1790	RNA processing	177	6.76E-04	cellular metabolic process	1277	1.63E-02
Grey60	102	83	positive regulation of cellular metabolic process	54	3.51E-05	cellular metabolic process	72	7.50E-03
Light Cyan	132	-	-	-	-	-	-	-
Light Green	99	-	-	-	-	-	-	-
Light Yellow	97	-	-	-	-	-	-	-

Magenta	290	246	microtubule polymerization or depolymerization	18	1.41E-03	positive regulation of biological process	158	4.81E-02
Midnight Blue	158	141	cotranslational protein targeting to membrane	18	1.50E-17	organonitrogen compound biosynthetic process	44	3.62E-05
Pink	314	277	immune response	130	5.50E-18	metabolic process	218	2.69E-02
Purple*	238	210	mitotic cell cycle process	44	1.50E-02	mitotic cell cycle	51	1.50E-02
Red*	409	377	biological adhesion	116	6.22E-10	biological regulation	322	1.73E-03
Royal Blue	97	96	organelle organization	54	2.15E-03	metabolic process	85	9.01E-03
Salmon	198	167	nucleosome assembly	12	1.87E-03	cellular protein-containing complex assembly	34	1.68E-02
Tan	224	-	-	-	-	-	-	-
Turquoise*	3769	3406	small molecule metabolic process	906	2.58E-69	metabolic process	2657	1.02E-28
Yellow*	1900	1563	metabolic process	1188	4.64E-05	metabolic process	1188	4.64E-05

Supplementary Table 2. Liver modules of co-expressed genes. See Supplementary Table 1 legend.

Liver Module	# of genes	# of annotated genes	Most Significant GO Term			Largest GO Term		
			GO term	Nr Test	FDR	GO term	Nr Test	FDR
Black	509	377	ncRNA metabolic process	47	1.13E-08	metabolic process	302	3.24E-03
Blue*	2848	2452	macromolecule metabolic process	1716	2.23E-26	cellular process	2204	1.11E-02
Brown*	1827	1645	ribonucleoprotein complex biogenesis	177	1.45E-43	metabolic process	1324	1.79E-21
Green	962	860	regulation of signal transduction	342	8.09E-04	metabolic process	657	3.22E-03
Green Yellow	156	129	cell development	62	2.36E-05	anatomical structure development	86	3.09E-02
Grey (unassigned)	2393	1903	-	-	-	-	-	-
Magenta	372	324	immune response	156	1.02E-23	cellular process	304	7.29E-03
Pink	409	391	intracellular transport	109	8.27E-05	organelle organization	166	1.73E-02
Purple	174	171	muscle filament sliding	23	3.73E-20	biological regulation	151	9.08E-03
Red*	937	846	immune response	312	2.32E-23	regulation of biological process	670	1.26E-02
Salmon	60	-	-	-	-	-	-	-
Tan	115	100	ribosome biogenesis	15	3.55E-05	ribonucleoprotein complex biogenesis	17	2.94E-04
Turquoise*	4014	3584	small molecule metabolic process	947	3.53E-71	cellular process	3206	1.31E-02
Yellow*	1175	1028	nucleic acid metabolic process	540	2.37E-24	metabolic process	800	5.87E-07

Supplementary Table 3. Muscle modules of co-expressed genes. See Supplementary Table 1 legend.

Muscle Module	# of genes	# of annotated genes	Most Significant GO Term			Largest GO Term		
			Go Term	Nr Test	FDR	GO Term	Nr Test	FDR
Black*	521	488	organic acid biosynthetic process	41	3.81E-04	metabolic process	388	2.35E-03
Blue*	2152	2013	mitochondrial gene expression	115	2.74E-61	metabolic process	1590	8.90E-20
Brown	1274	1213	metabolic process	950	1.34E-08	metabolic process	950	1.34E-08
Cyan*	234	-	-	-	-	-	-	-
Dark Green	132	130	protein demethylation	8	2.79E-04	macromolecule metabolic process	99	3.06E-02
Dark Grey	103	-	-	-	-	-	-	-
Dark Orange	90	87	cellular response to organonitrogen compound	27	1.20E-05	organic substance metabolic process	74	2.10E-02
Dark Red	134	131	maintenance of cell polarity	7	1.36E-03	maintenance of cell polarity	7	1.36E-03
Dark Turquoise*	120	114	-	-	-	-	-	-
Green*	800	688	cotranslational protein targeting to membrane	43	2.96E-34	metabolic process	546	2.87E-06
Green Yellow	374	346	actin-mediated cell contraction	36	6.60E-11	metabolic process	271	1.22E-02
Grey (unassigned)	764	670	ribonucleoprotein complex biogenesis	58	2.09E-07	metabolic process	513	2.77E-02
Grey 60	222	203	intracellular protein transmembrane transport	10	3.88E-05	ncRNA metabolic process	20	4.48E-02
Light Cyan*	230	221	cellular localization	94	6.34E-03	cellular localization	94	6.34E-03
Light Green	177	169	protein metabolic process	107	9.84E-03	macromolecule metabolic process	127	4.25E-02
Light Yellow	168	160	mRNA metabolic process	31	3.93E-05	macromolecule metabolic process	119	2.03E-02
Magenta	400	374	adaptive immune response	66	6.55E-23	response to stimulus	280	1.96E-02
Midnight Blue*	232	218	transcription by RNA polymerase II	95	1.30E-11	metabolic process	178	5.59E-03
Orange*	92	89	extracellular matrix organization	14	3.22E-03	extracellular matrix organization	14	3.22E-03
Pink	405	391	regulation of gene expression	219	2.64E-14	metabolic process	322	2.55E-06
Purple	397	384	cell adhesion	138	5.14E-19	biological regulation	323	5.66E-03
Red*	578	522	nucleic acid metabolic process	253	1.98E-05	organic substance metabolic process	391	2.55E-02

Royal Blue*	147	135	negative regulation of nucleobase-containing compound metabolic process	42	1.92E-02	negative regulation of gene expression	45	4.96E-02
Saddle Brown	71	-	-	-	-	-	-	-
Salmon	253	-	-	-	-	-	-	-
Sky Blue*	74	-	-	-	-	-	-	-
Steel Blue	65	-	-	-	-	-	-	-
Tan*	360	-	-	-	-	-	-	-
Turquoise	2407	2344	multicellular organism development	1543	2.18E-83	cellular process	2159	2.34E-11
White	81	-	-	-	-	-	-	-
Yellow*	1035	962	nucleic acid metabolic process	512	8.92E-25	metabolic process	766	4.19E-10

Supplementary Table 4. Overlap between this study and genes that responded to clinical treatment for loss of insulin sensitivity [3]. Genes that were differentially expressed in our study are indicated with an arrow that also indicates direction of change in the hibernation vs non-hibernation state comparison.

Gene name	Db-xref Number	Gene Annotation	DE Adipose	DE Liver	DE Muscle
gene6715	113254363	*AGL*	↓	↑	↓
gene14200	113262646	*G0S2*	↓		↑
gene16598	113265264	*KPNA2*			
gene13983	113269311	*PGM2*		↓	
gene3709	113250895	*RND3*	↑	↑	
gene10094	113258011	*TSPAN9*	↓	↓	↓
gene21081	113270345	*ALDH6A1*	↓	↓	↓
gene22418	113241065	*DHTKD1*		↓	↓
gene22413	113241060	*ECHDC3*	↓		↓
gene834	113254564	*MCCC1*	↓	↓	↓
gene13108	113261321	*OARD1*	↓		↓
gene25484	113244495	*PCYT2*	↓	↓	↓
gene14450	113262848	*PRRX1*	↑		
gene3785	113251183	*SGCG*	↓		
gene27295	113246534	*SLC43A1*		↓	
gene16258	113264847	*SMIM8*			

Supplementary Table 5. Body Weight (kg)

Bear	Jan	May	Sep
**J**	252	258	310
**F**	264	299	290
**C**	160	139	164
**O**	163	152	176
**R**	205	170	212
**P**	187	154	191

Supplementary Table 6. Body Temperature (°C)

Bear	Jan	May	Sep
**J**	34.4	37.2	35.8
**F**	34.8	36.8	36.8
**C**	32.6	37.1	36.4
**O**	34.9	35.9	35.5
**R**	32.9	37.2	36.5
**P**	34.2	36.9	37.2

References

1 Hu, Z., Snitkin, E. S. & DeLisi, C. VisANT: an integrative framework for networks in systems biology. *Brief Bioinform* **9**, 317-325, doi:10.1093/bib/bbn020 (2008).

2 Fedorov, V. B. *et al.* Modulation of gene expression in heart and liver of hibernating black bears (*Ursus americanus*). *Bmc Genomics* **12** (2011).

3 Timmons, J. A. *et al.* A coding and non-coding transcriptomic perspective on the genomics of human metabolic disease. *Nucleic Acids Res*, doi:10.1093/nar/gky570 (2018).
