# Supplementary material for: Hibernation induces widespread transcriptional remodeling in metabolic tissues of the grizzly bear
Source: Commun Biol. 2019 Sep 13;2:336. doi: 10.1038/s42003-019-0574-4 (PMC6744400; doi:10.1038/s42003-019-0574-4)
Supplement: Supplementary file 2 — Description of Additional Supplementary Files [file 42003_2019_574_MOESM2_ESM.pdf]

Description of additional supplementary items for

Hibernation induces widespread transcriptional remodeling in metabolic tissues of the grizzly bear

Description of additional supplementary items:

Supplementary Data 1 to 7, which are included as separate excel files

Supplementary Data

Supplementary Data 1. Data summary

Total number of reads, mapped reads and percent mapped per sample. Sample ID includes animal ID (first letter), tissue (second letter; A-adipose, L-liver, M-muscle) and season (third and fourth letters; A-active, Hy-hyperphagia, Hi-hibernation). Provided as separate excel file.

Supplementary Data 2. Summary of all genes

Summary of expression data and glmQLFTest for each gene in all three tissues. Log_2 fold change (logFC), log counts per million (logCPM), F-test statistic (F), Pvalue, and false discovery rate (FDR) are reported for each gene in each tissue. Annotation from the bear genome (Annotation) and top BLAST hit in the human SWISSPROT database (Description) are also reported. The median counts per million (medianCPM) are reported for each tissue. Provided as separate excel file.

Supplementary Data 3. Enriched gene ontology terms

Gene ontology terms that are enriched in differentially expressed genes from each tissue. Downregulated and upregulated genes were analyzed separately. GO categories are highlighted in each tab: Biological Process (blue), Cellular Component (green), and Molecular Function (yellow). Provided as separate excel file.

Supplementary Data 4. Gene set enrichment analysis.

Gene set enrichment analyses with the curated gene set (C2) and the GO biological process gene sets. Results are reported on separate tabs for each tissue and gene set. The enrichment score

(ES) was calculated within GSEA and reflects whether the gene set is overrepresented at the top or bottom of the ranked list. The normalized enrichment score (NES) accounts for differences in gene set size. Provided as separate excel file.

Supplementary Data 5. Gene ontology terms from intersection of all three tissues

Gene ontology terms that are enriched in differentially expressed genes from the intersection of genes that were differentially expressed in all tissues. GO categories are highlighted in each tab: Biological Process (blue), Cellular Component (green), and Molecular Function (yellow). Provided as separate excel file.

Supplementary Data 6. Module assignment for the tissue specific WGCNA.

Gene name, gene symbol, and module color are reported for each tissue. Provided as separate excel file.

Supplementary Data 7. Genes that are differentially expressed between active and hyperphagia

Summary of expression data and glmQLFTest for each gene in all three tissues. Log_2 fold change (logFC), log counts per million (logCPM), F-test statistic (F), p-value, and FDR are reported for each gene in each tissue. Annotation from the bear genome (Existing_Annotation) and top BLAST hit in human SWISSPROT (TopBLASThit) are also reported. Provided as separate excel file.
