# Supplementary material for: Hibernation induces widespread transcriptional remodeling in metabolic tissues of the grizzly bear
Source: Commun Biol. 2019 Sep 13;2:336. doi: 10.1038/s42003-019-0574-4 (PMC6744400; doi:10.1038/s42003-019-0574-4)
Supplement: Supplementary file 10 — Reporting Summary [file 42003_2019_574_MOESM10_ESM.pdf]

natureresearch

Corresponding author(s): Jansen, Kelley

Last updated by author(s): 07/23/2019

Reporting Summary

Nature Research wishes to improve the reproducibility of the work that we publish. This form provides structure for consistency and transparency in reporting. For further information on Nature Research policies, seeAuthors & Referees and theEditorial Policy Checklist .

Statistics

For all statistical analyses, confirm that the following items are present in the figure legend, table legend, main text, or Methods section.

n/a	Confirmed	
☐	☒	The exact sample size (*n*) for each experimental group/condition, given as a discrete number and unit of measurement
☐	☒	A statement on whether measurements were taken from distinct samples or whether the same sample was measured repeatedly
☐	☒	The statistical test(s) used AND whether they are one- or two-sided<br>*Only common tests should be described solely by name; describe more complex techniques in the Methods section.*
☒	☐	A description of all covariates tested
☐	☒	A description of any assumptions or corrections, such as tests of normality and adjustment for multiple comparisons
☐	☒	A full description of the statistical parameters including central tendency (e.g. means) or other basic estimates (e.g. regression coefficient) AND variation (e.g. standard deviation) or associated estimates of uncertainty (e.g. confidence intervals)
☐	☒	For null hypothesis testing, the test statistic (e.g. *F*, *t*, *r*) with confidence intervals, effect sizes, degrees of freedom and *P* value noted<br>*Give P values as exact values whenever suitable.*
☒	☐	For Bayesian analysis, information on the choice of priors and Markov chain Monte Carlo settings
☒	☐	For hierarchical and complex designs, identification of the appropriate level for tests and full reporting of outcomes
☒	☐	Estimates of effect sizes (e.g. Cohen's *d*, Pearson's *r*), indicating how they were calculated

Our web collection on statistics for biologists contains articles on many of the points above.

Software and code

Policy information about availability of computer code

Data collection	No software was used for data collection.
Data analysis	Raw sequencing reads were assessed with FastQC version 0.11.4. Reads were trimmed using Trim Galore! (Cutadapt version 0.4.2). Trimmed reads were mapped to the brown bear reference genome assembly using HISAT2 (version 2.1.0). Mate pair information was verified and fixed where necessary using Picard tools (version 2.2.1). Output SAM files were converted to BAM format and sorted by coordinates with SAMtools (version 1.2). Stringtie (version 1.3.4d) was used to estimate the number of reads mapping to each gene in the reference annotation set of the brown bear genome (NCBI Ursus arctos horribilis Annotation Release 100). All coding genes were annotated using a BLASTx search (version 2.2.31) against the human SwissProt database (access date 6/9/2018). Blast2GO (version 5.2.0) was used to explore the putative biological functions of candidate gene sets. Data were transformed using the variance-stabilizing transformation function in the DESEQ2 (version 1.22.2) package in R (version 3.5.2) prior to plotting. The limma package (version 3.38.3) in R was used to plot MDS. Differential expression was quantified based on normalized read counts using the Bioconductor package edgeR (version 3.24.3). Gene set enrichment analysis was performed in GSEA (version 3.0). Weighted gene co-expression network analysis was conducted using R package WGCNA (version 1.66). All code is available at https://github.com/jokelley/brownbear-rnaseq-tissue-act-hyp-hib

For manuscripts utilizing custom algorithms or software that are central to the research but not yet described in published literature, software must be made available to editors/reviewers. We strongly encourage code deposition in a community repository (e.g. GitHub). See the Nature Research guidelines for submitting code & software for further information.

Data

Policy information about availability of data

All manuscripts must include a data availability statement. This statement should provide the following information, where applicable:

- Accession codes, unique identifiers, or web links for publicly available datasets
- A list of figures that have associated raw data
- A description of any restrictions on data availability

All data are available in Genbank BioProject PRJNA413091.

Field-specific reporting

Please select the one below that is the best fit for your research. If you are not sure, read the appropriate sections before making your selection.

☒ Life sciences ☐ Behavioural & social sciences ☐ Ecological, evolutionary & environmental sciences

For a reference copy of the document with all sections, see nature.com/documents/nr-reporting-summary-flat.pdf

Life sciences study design

All studies must disclose on these points even when the disclosure is negative.

Sample size	Sample size was determined by the availability of samples. All available samples were used resulting in a sample size of 6 bears. An N=6 has been shown to be appropriate by simulation studies (Todd, E. V., Black, M. A., & Gemmell, N. J. (2016). The power and promise of RNA-seq in ecology and evolution. Molecular ecology, 25(6), 1224-1241.)
Data exclusions	One sample (CAA) was removed from analyses because the expression analysis revealed that a hair follicle was inadvertently sampled with the adipose tissue. This was determined based on the expression profile of that sample. This was not a pre-established criteria.
Replication	We sampled two of the bears the following winter to measure the correlation between years.
Randomization	Not relevant as there were no experimental groups
Blinding	Blinding was not possible for this experiment as researchers were present for the sampling of the 6 bears.

Reporting for specific materials, systems and methods

We require information from authors about some types of materials, experimental systems and methods used in many studies. Here, indicate whether each material, system or method listed is relevant to your study. If you are not sure if a list item applies to your research, read the appropriate section before selecting a response.

Materials & experimental systems

n/a	Involved in the study	
☒	☐	Antibodies
☒	☐	Eukaryotic cell lines
☒	☐	Palaeontology
☐	☒	Animals and other organisms
☒	☐	Human research participants
☒	☐	Clinical data

Methods

n/a	Involved in the study	
☒	☐	ChIP-seq
☒	☐	Flow cytometry
☒	☐	MRI-based neuroimaging

Animals and other organisms

Policy information about studies involving animals; ARRIVE guidelines recommended for reporting animal research

Laboratory animals	All animals were grizzly bear (Ursus arctos horribilis). Ages of animals ranged from 5 years (R, P) to approximately 14 years (F, J, C, O). F and J are at least half siblings, C and O are unrelated, and R and P are related and the offspring of F, J, C, and O. Four males were used for this study (R, P, F, J) and two females (C, O).
Wild animals	The study did not involve wild animals
Field-collected samples	The study did not involve field-collected samples
Ethics oversight	All protocols were approved by the Washington State University Institutional Animal Care and Use Committee (IACUC). Protocol #03875 and #04922.

Note that full information on the approval of the study protocol must also be provided in the manuscript.
